# Supplementary material for: Hyperglycaemia inhibits REG3A expression to exacerbate TLR3-mediated skin inflammation in diabetes
Source: Nat Commun. 2016 Nov 10;7:13393. doi: 10.1038/ncomms13393 (PMC5109591; doi:10.1038/ncomms13393)
Supplement: Supplementary Information — Supplementary Figures 1-14, Supplementary Tables 1-5 and Supplementary Methods. [file ncomms13393-s1.pdf]

Supplementary Figures

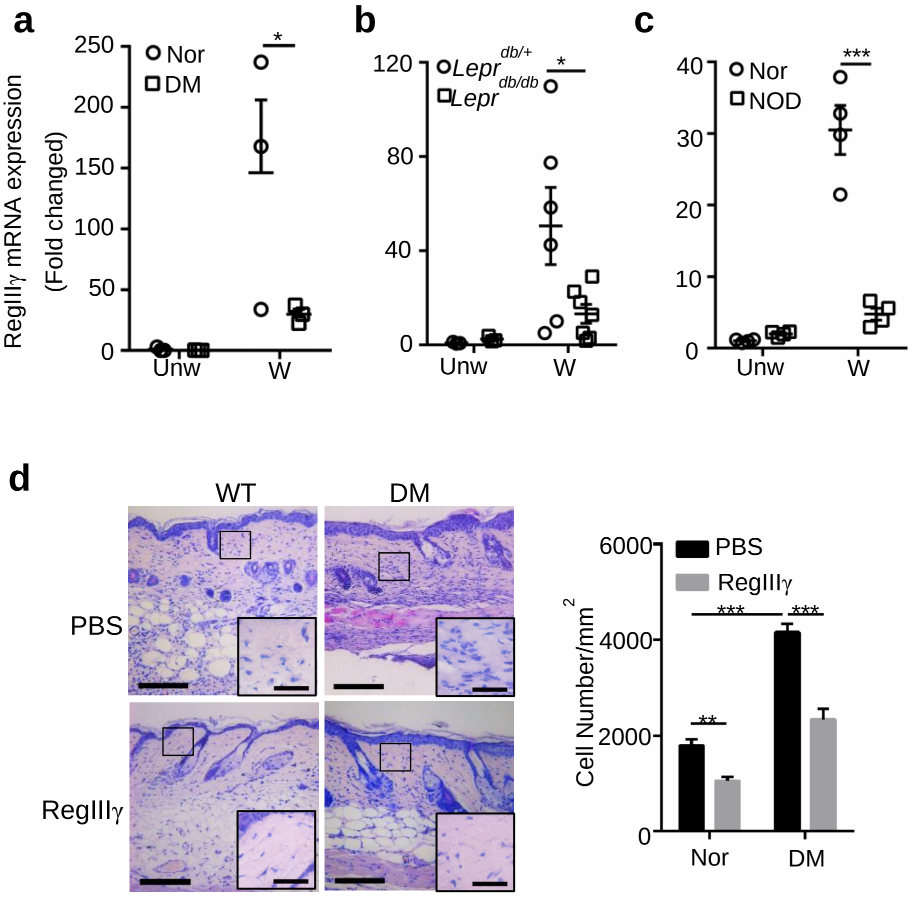

Supplementary Figure 1. Skin injury increases RegIIIγ expression to regulate inflammatory responses. (a) Quantification of RegIIIγ mRNA expression in day-3 skin wounds of BALB/c normal and STZ-induced type 1 diabetic mice (*n*=3). **(b)** Quantification of RegIIIγ mRNA expression in day-3 skin wounds of $Lepr^{db/+}$ (*n*=6) and $Lepr^{db/db}$ mice (*n*=7). **(c)** Quantification of RegIIIγ mRNA expression in day-3 skin wounds of normal and NOD mice (*n*=4). **(d)** H&E staining of skin 2mm adjacent to day-3 skin wounds of normal and T1D mice (left) (*n*=4). Long scale bars represent 200μm, and short scale bars represent 50μm. Black rectangles designate region of 400× magnification shown in insets. Leukocytes in per 1mm^2 dermis were counted and the mean value was calculated (right). **P*<0.05, ***P*<0.01, ****P*<0.001. *P* values were analyzed by Two-way ANOVA. Data are the means ± s.e.m and representative of two independent experiments.

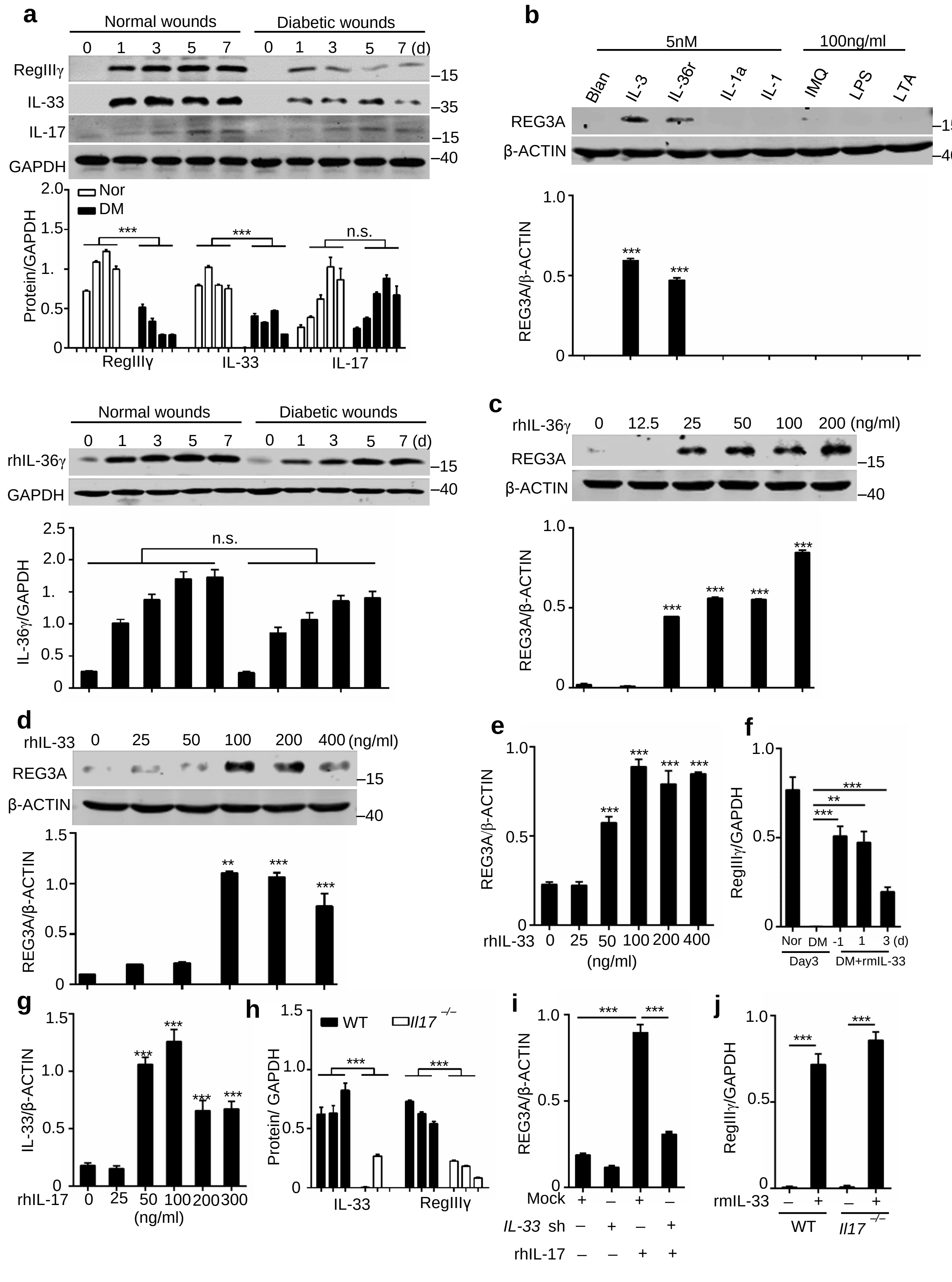
a
Normal wounds
Diabetic wounds
0 1 3 5 7 0 1 3 5 7 (d)
RegIIIγ
IL-33
IL-17
GAPDH
–15
–35
–15
–40
Nor
DM
Protein/GAPDH
***
***
n.s.
RegIIIγ
IL-33
IL-17
Normal wounds
Diabetic wounds
rhIL-36γ
GAPDH
IL-36γ/GAPDH
n.s.
b
5nM
100ng/ml
Blan
IL-3
IL-36r
IL-1a
IL-1
IMQ
LPS
LTA
REG3A
β-ACTIN
REG3A/β-ACTIN
c
rhIL-36γ
0 12.5 25 50 100 200 (ng/ml)
REG3A
β-ACTIN
REG3A/β-ACTIN
d
rhIL-33
0 25 50 100 200 400 (ng/ml)
REG3A
β-ACTIN
REG3A/β-ACTIN
e
REG3A/β-ACTIN
rhIL-33 0 25 50 100 200 400
(ng/ml)
f
RegIIIγ/GAPDH
Nor DM -1 1 3 (d)
Day3
DM+rmIL-33
g
IL-33/β-ACTIN
rhIL-17 0 25 50 100 200 300
(ng/ml)
h
WT
Il17 –/–
Protein/ GAPDH
IL-33
RegIIIγ
i
REG3A/β-ACTIN
Mock
IL-33 sh
rhIL-17
j
RegIIIγ/GAPDH
rmIL-33
WT
Il17 –/–

Supplementary Figure 2. IL-33 and IL-36γ regulate REG3A expression. (a) Immunoblot of RegIIIγ, IL-33, IL-17 and IL-36γ in skin extracts taken from 2 mm surrounding the wound edges of normal and T1D mice at indicated times. (**b**) Immunoblot of REG3A in NHEKs treated with 5nM IL-33, IL-36γ, IL-1α, IL-18, or 100ng/ml Imiquimod (IMQ), LPS, LTA. **(c)** Immunoblot of REG3A in NHEKs stimulated by different doses of rhIL-36γ for 12h. (**d**) Immunoblot of REG3A in AHEKs stimulated by different doses of rhIL-33 for 12h. (**e**) The densitometry of the bands of REG3A corresponding to Fig.2b. (**f**) The densitometry of the bands of RegIIIγ corresponding to Fig.2f. (**g**) The densitometry of the bands of IL-33 corresponding to Fig.2h. (**h**) The densitometry of the bands of IL-33 and RegIIIγ corresponding to Fig.2i. (**i**) The densitometry of the bands of REG3A corresponding to Fig.2j. (**j**) The densitometry of the bands of RegIIIγ corresponding to Fig.2k. The densitometry of all the bands was analyzed by Image J and normalized to β-ACTIN or GAPDH. ***P<0.001. n.s. no significance. P values were analyzed by Two-way ANOVA (**a, h**) or One-way ANOVA (**b-g, i, j**). Data are the means ± s.e.m and representative of two-three independent experiments.

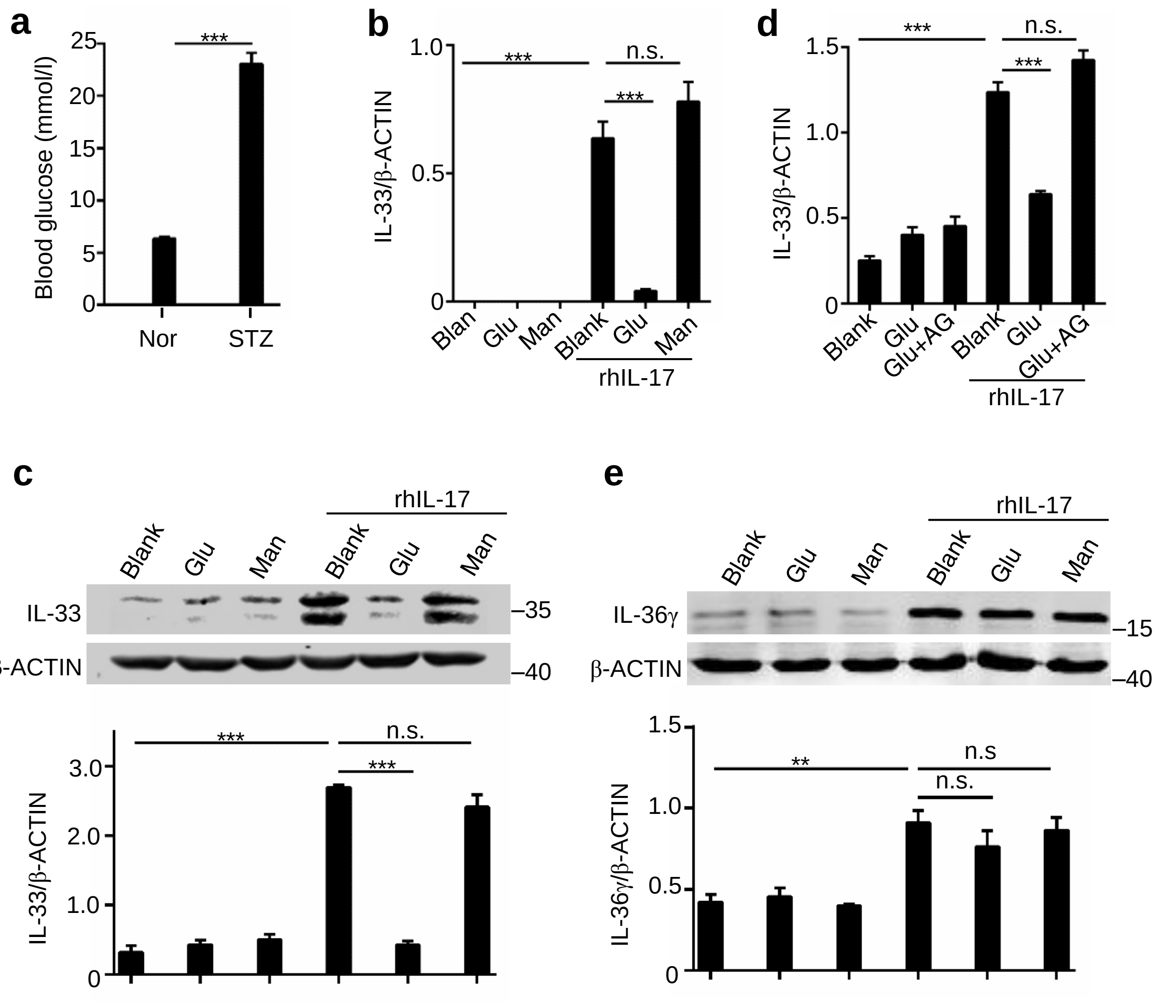

Supplementary Figure 3. High glucose inhibits the expression of IL-17-induced IL-33 but not IL-36γ. (a) The concentrations of blood glucose in T1D mice (*n*=8). (**b**) The densitometry of the bands of IL-33 corresponding to Fig.2l. **(c)** IL-33 production induced by 200ng ml^{-1} rhIL-17 in AHEKs exposed to 20mM glucose or mannitol for 24h. (**d**) The densitometry of the bands of IL-33 corresponding to Fig.2n. (**e**) IL-36γ production in NHEKs treated as in (**c**). The densitometry of all the bands was analyzed by Image J and normalized to β-ACTIN. ** P<0.01 and ***P<0.001. n.s. no significance. P values were analyzed by Two-tailed t tests **(a)** or One-way ANOVA **(b-e)**. Data are the means ± s.e.m and representative of two-three independent experiments.

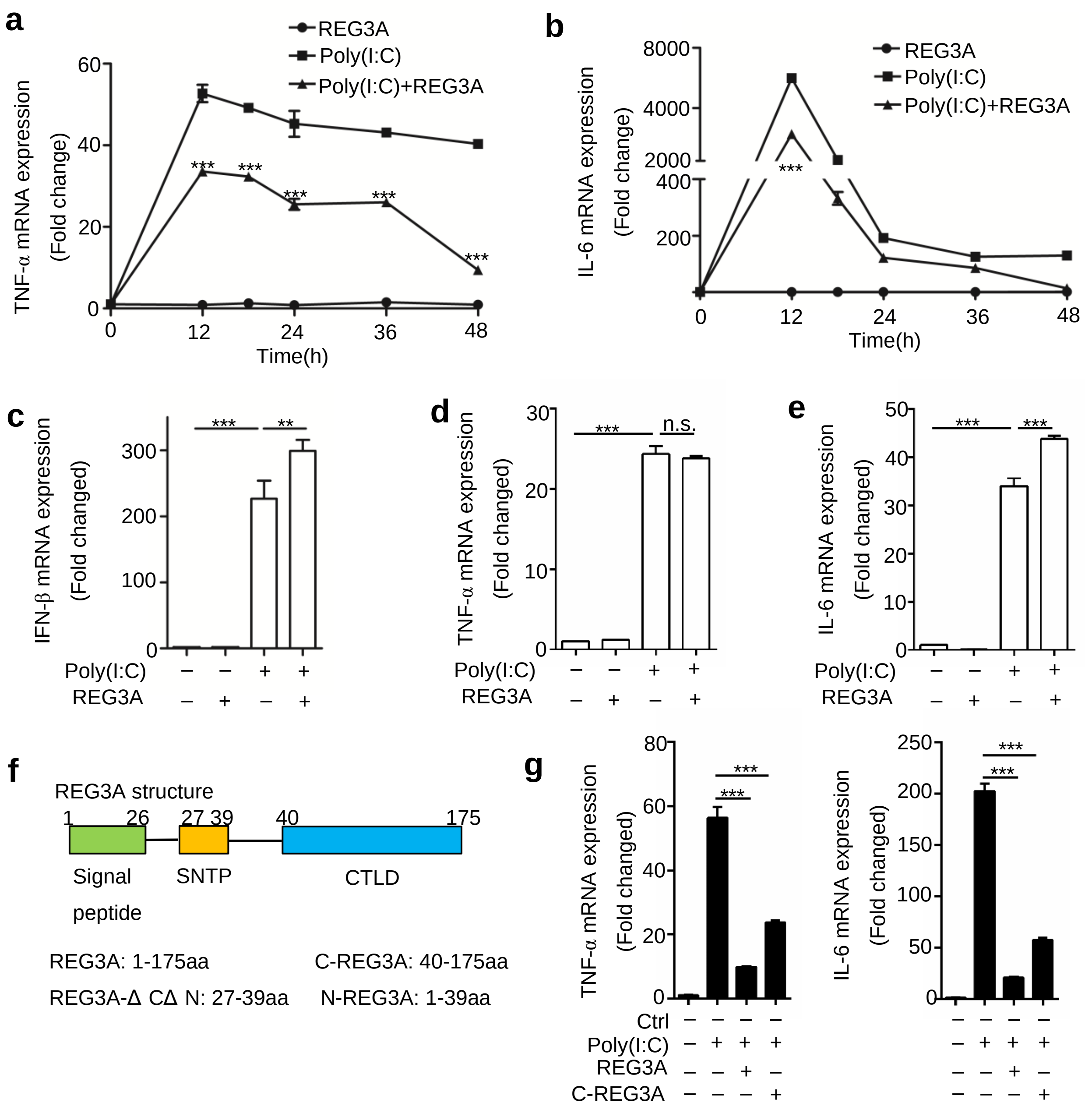

Supplementary Figure 4. REG3A regulates TLR3-mediated inflammatory response. (**a&b**) Quantification of TNF-α (**a**) and IL-6 (**b**) mRNA expression in NHEKs treated with 5μg ml^{-1} poly(I:C) in the presence or absence of 30nM REG3A for indicated times (*n*=3). (**c**) Quantification of IFN-β mRNA expression in NHEKs treated with 5μg ml^{-1} poly(I:C) in the absence or presence of 30nM REG3A for 24h (*n*=3). (**d&e**) Quantification of TNF-α (**d**) and IL-6 (**e**) mRNA expression in THP-1 cells treated as in (**c**) (*n*=3). (**f**) Schematic overview of different domains of REG3A. (**g**) Quantification of TNF-α and IL-6 mRNA expression in AHEKs treated with 10μg ml^{-1} poly(I:C) in the absence or presence of 30nM

REG3A or C-REG3A (*n*=3). $^{**}P<0.01$ and $^{***}P<0.001$. n.s. no significance. *P* values were determined by Two-way ANOVA in (**a&b**) or One-way ANOVA in (**c-e, and g**). Data are the means ±s.e.m and representative of two to three independent experiments.

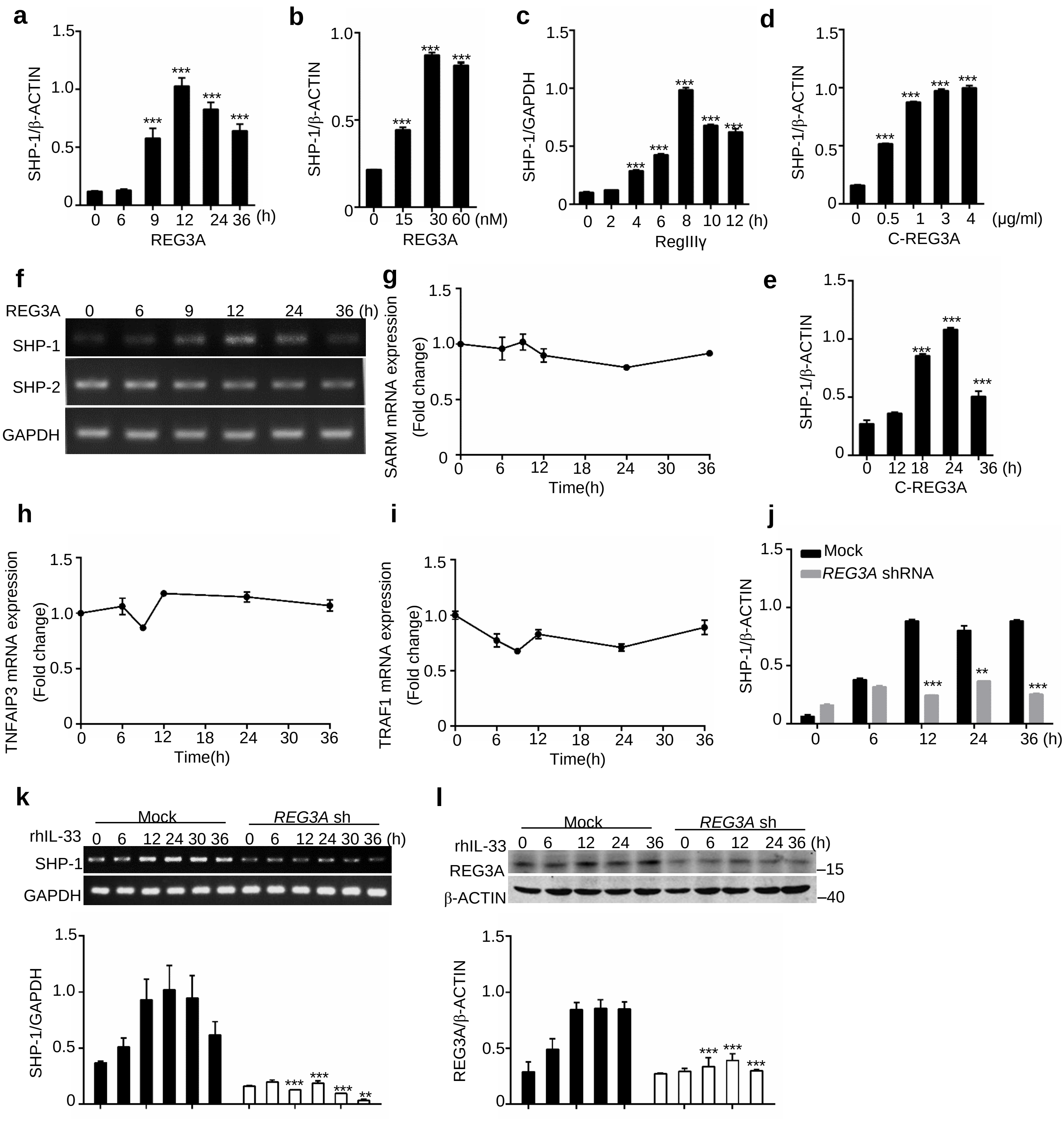

a
SHP-1/β-ACTIN
REG3A
0 6 9 12 24 36 (h)
b
SHP-1/β-ACTIN
REG3A
0 15 30 60 (nM)
c
SHP-1/GAPDH
RegIIIγ
0 2 4 6 8 10 12 (h)
d
SHP-1/β-ACTIN
C-REG3A
0 0.5 1 3 4 (μg/ml)
f
REG3A 0 6 9 12 24 36 (h)
SHP-1
SHP-2
GAPDH
g
SARM mRNA expression (Fold change)
Time(h)
e
SHP-1/β-ACTIN
C-REG3A
0 12 18 24 36 (h)
h
TNFAIP3 mRNA expression (Fold change)
Time(h)
i
TRAF1 mRNA expression (Fold change)
Time(h)
j
Mock
REG3A shRNA
SHP-1/β-ACTIN
0 6 12 24 36 (h)
k
Mock
REG3A sh
rhIL-33 0 6 12 24 30 36 0 6 12 24 30 36 (h)
SHP-1
GAPDH
SHP-1/GAPDH
l
Mock
REG3A sh
rhIL-33 0 6 12 24 36 0 6 12 24 36 (h)
REG3A
β-ACTIN
–15
–40
REG3A/β-ACTIN

Supplementary Figure 5. REG3A activates EXTL3 to induce SHP-1 in keratinocytes. **(a)** The densitometry of the bands of SHP-1 corresponding to Fig.4a. (**b**) The densitometry of the bands of SHP-1 corresponding to Fig.4b. (**c**) The densitometry of the bands of SHP-1 corresponding to Fig.4c. (**d**) The densitometry of the bands of SHP-1 corresponding to Fig.4d. (**e**) The densitometry of the bands of SHP-1 corresponding to Fig.4e. (**f**) RT-PCR of SHP-1 and SHP-2 mRNA expression in NHEKs stimulated by 30nM REG3A for indicated times. (**g-i**) Quantification of SARM (**g**), TNFAIP3 (**h**) and TRAF1 (**i**) mRNA expression by qPCR in NHEKs treated as in (**f**) (n=3). (**j**) The densitometry of the bands of SHP-1 corresponding to Fig.4f. (**k**) RT-PCR of SHP-1 mRNA expression in NHEKs stimulated by 100ng ml^{-1} rhIL-33 for indicated times before and after REG3A silencing. (**l**) Immunoblot of REG3A in NHEKs stimulated by rhIL-33 for indicated times before and after REG3A silencing. The densitometry of all the bands was analyzed by Image J and normalized to β-ACTIN or GAPDH. ** P<0.01 and ***P<0.001. P values were analyzed by One-way ANOVA (**a-e**) or Two-way ANOVA (**j-l**). Data are the means ± s.e.m. and representative of three independent experiments.

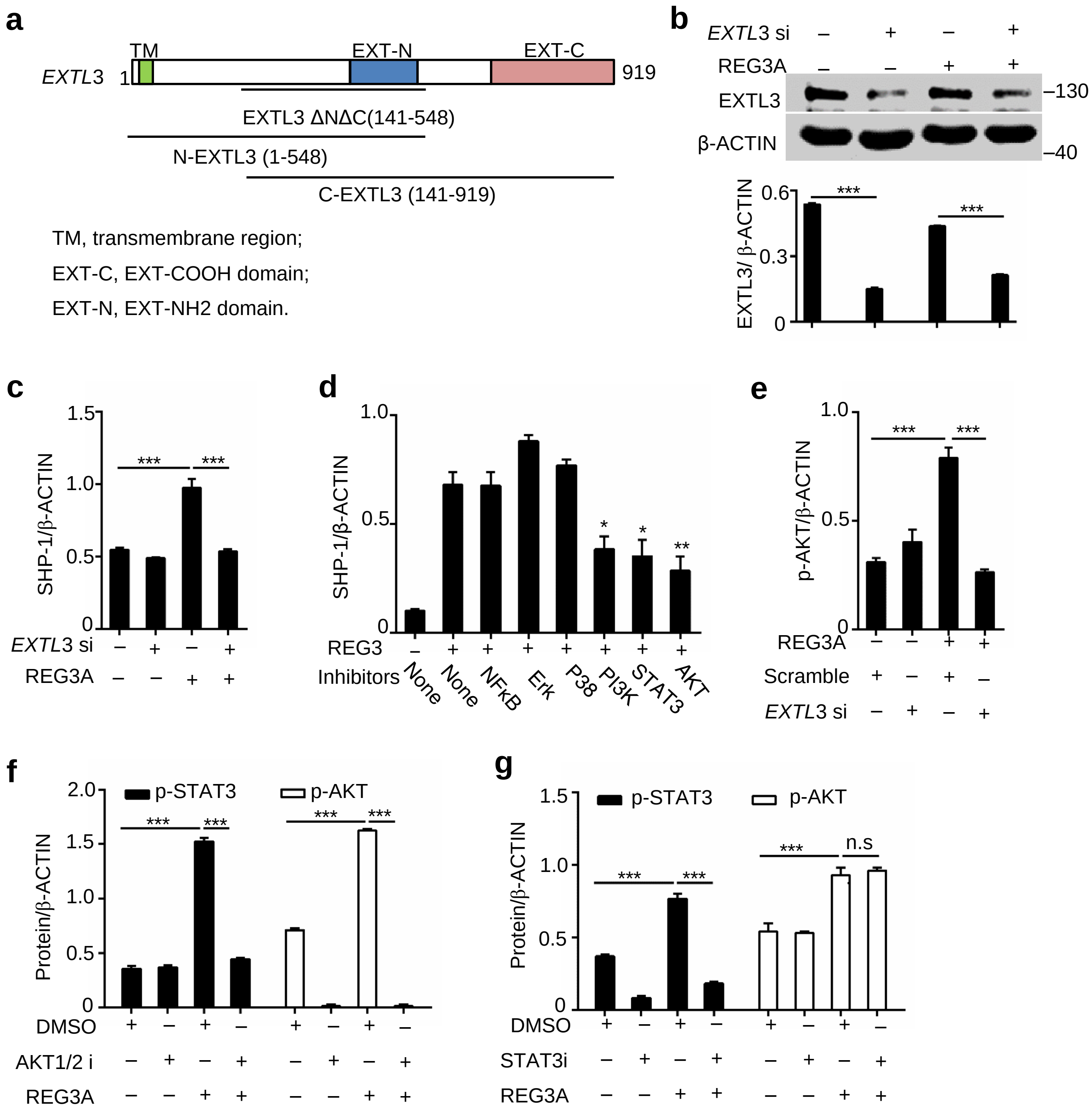

Supplementary Figure 6. REG3A activates EXTL3-PI3K-AKT-STAT3 in the induction of SHP-1 in keratinocytes. (a) Schematic overview of different domains of EXTL3. (**b**) Immunoblot of EXTL3 in NHEKs treated with 30nM REG3A before and after EXTL3 silencing. (**c**) The densitometry of the bands of SHP-1 corresponding to Fig.5d. (**d**) The densitometry of the bands of SHP-1 corresponding to Fig.5e. (**e**) The densitometry of the bands of p-AKT corresponding to Fig.5f. (**f**) The densitometry of the bands of p-STAT3 and

p-AKT corresponding to Fig.5g. (**g**) The densitometry of the bands of p-STAT3 and p-AKT corresponding to Fig.5h. The densitometry of all the bands was analyzed by Image J and normalized to β-ACTIN. *P<0.05, ** P<0.01 and ***P<0.001. n.s. no significance. P values were analyzed by One-way ANOVA. Data are the means ± s.e.m. and representative of two to three independent experiments.

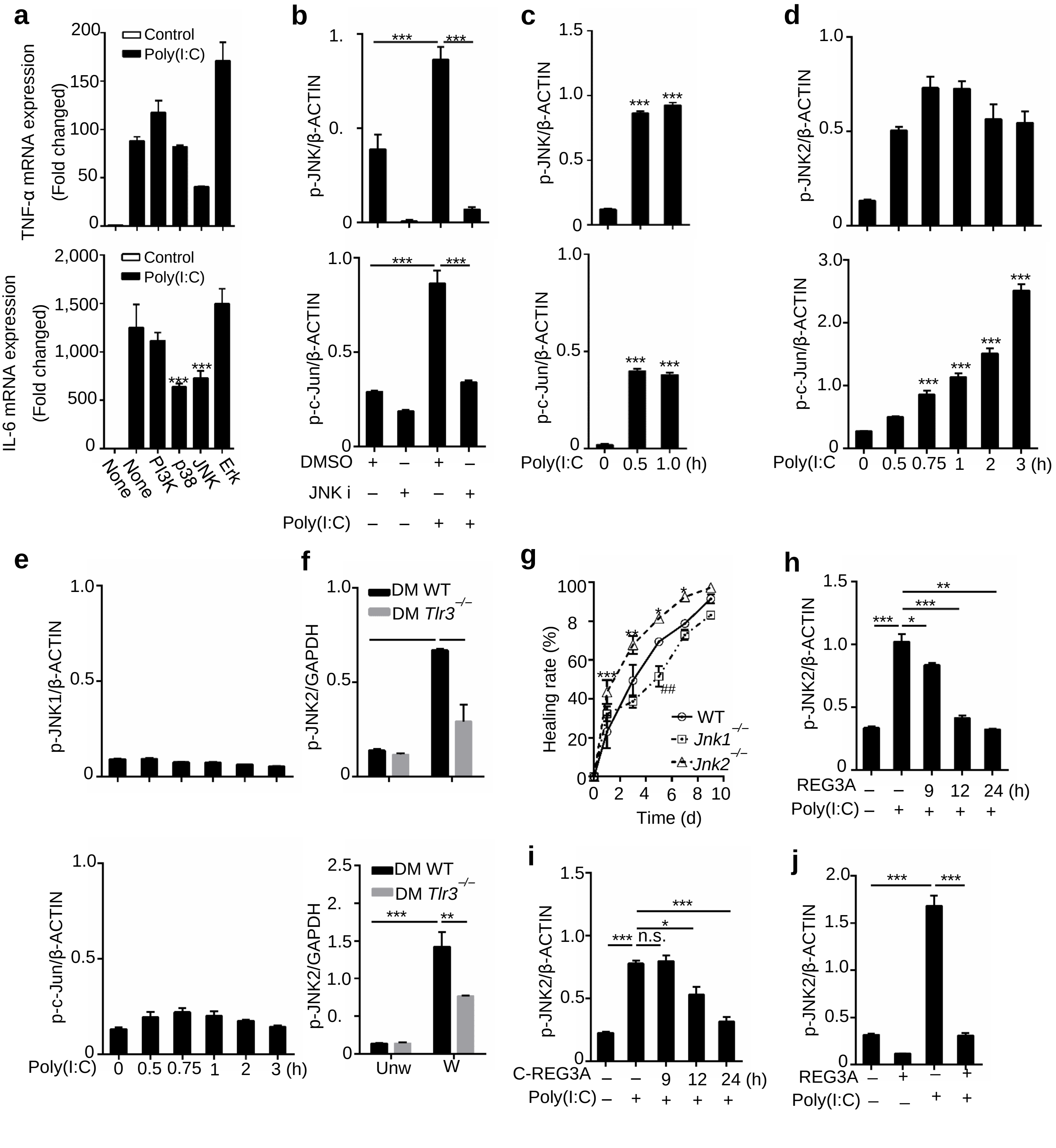

Supplementary Figure 7. The induction of TNF-α and IL-6 is dependent on JNK activation in keratinocytes and in skin wounds. (**a**)The mRNA expression of TNF-α and IL-6 was quantified by qPCR in NHEKs treated with 5μg ml^{-1} poly(I:C) in the presence or

absence of p38 MAPK inhibitor (SB202190, 5μM), PI3K inhibitor (Wortmanin, 5μM), JNK inhibitor (SP600125, 15μM) and Erk inhibitor (PD98059, 20μM) (*n*=3). (**b**) The densitometry of the bands of p-JNK and p-c-Jun corresponding to Fig.6c. (**c**) The densitometry of the bands of p-JNK and p-c-Jun corresponding to Fig.6f. (**d**) The densitometry of the bands of p-JNK2 and p-c-Jun corresponding to Fig.6g. (**e**) The densitometry of the bands of p-JNK1 and p-c-Jun corresponding to Fig.6h. (**f**) The densitometry of the bands of p-JNK2 corresponding to Fig.6i,j. (**g**) Wound healing of WT, *Jnk1*$^{-/-}$ and *Jnk2*$^{-/-}$ normal mice (*n*=6). (**h**) The densitometry of the bands of p-JNK2 corresponding to Fig.6m. (**i**) The densitometry of the bands of p-JNK2 corresponding to Fig.6n. (**j**) The densitometry of the bands of p-JNK2 corresponding to Fig.6o. The densitometry of all the bands was analyzed by Image J and normalized to β-ACTIN or GAPDH. *P<0.05, ** or ## P<0.01, ***P<0.001. P values were analyzed by One-way ANOVA (**a-e, h-j**) or Two-way ANOVA (**f,g**). Data are the means ± s.e.m. and representative of two to three independent experiments.

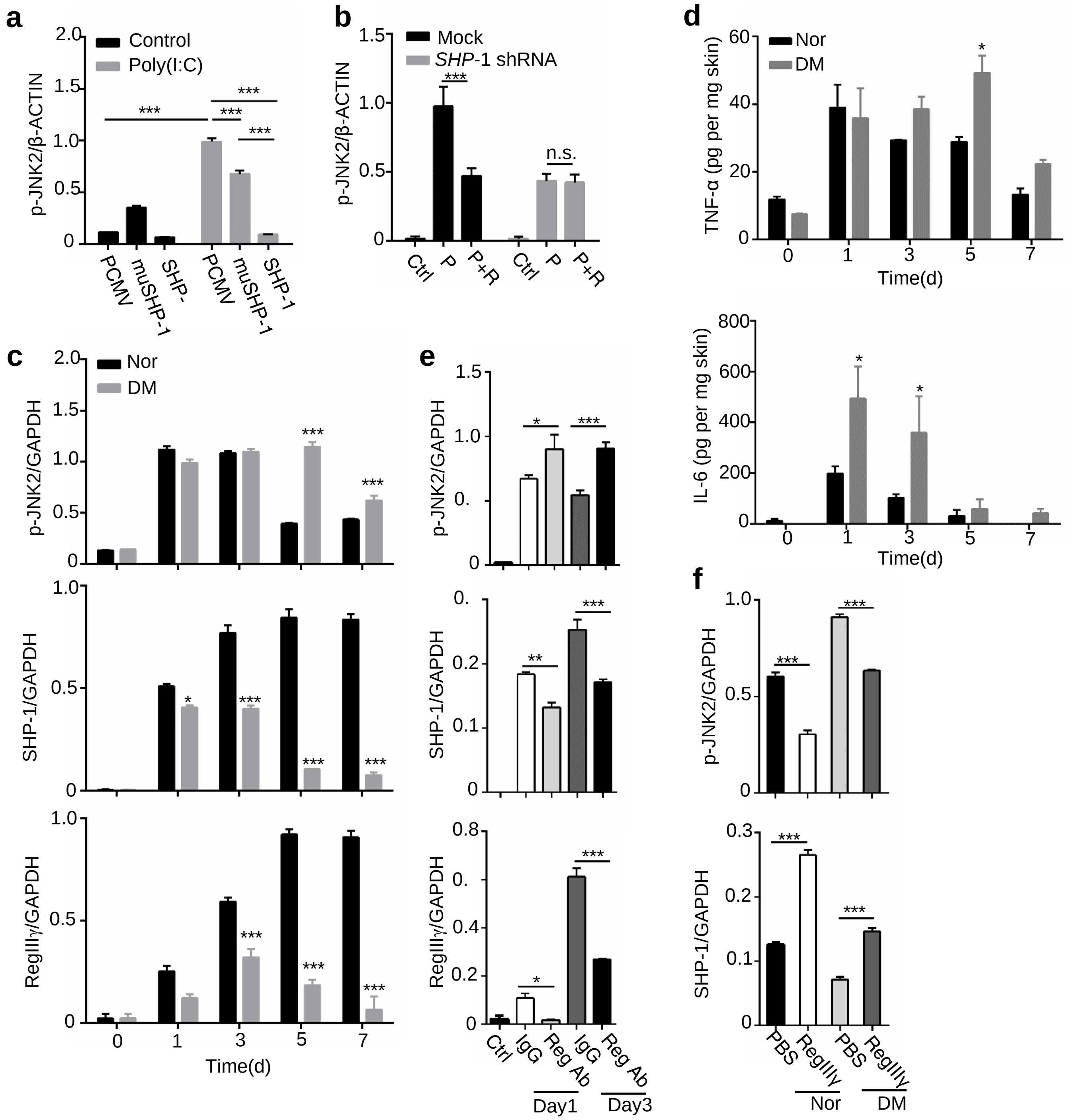

Supplementary Figure 8. SHP-1 induced by REG3A dephosphorylates phospho-JNK2. (**a**) The densitometry of the bands of p-JNK2 corresponding to Fig.7a. (**b**) The densitometry of the bands of p-JNK2 corresponding to Fig.7b. (**c**) The densitometry of the bands of p-JNK2, SHP-1 and RegIIIγ corresponding to Fig.7d. (**d**) The production of TNF-α and IL-6 in skin extracts taken from 2 mm surrounding the wound edges from normal and T1D mice at indicated times (*n*=3). (**e**) The densitometry of the bands of p-JNK2, SHP-1 and RegIIIγ

corresponding to Fig.7e. (**f**) The densitometry of the bands of p-JNK2 and SHP-1 corresponding to Fig.7f. The densitometry of all the bands was analyzed by Image J and normalized to β-ACTIN or GAPDH. *P<0.05, **P<0.01 and ***P<0.001. n.s. no significance. P values were determined by Two-way ANOVA (**a-e**) or One-way ANOVA (**f**). Data are the means ± s.e.m. and representative of two independent experiments.

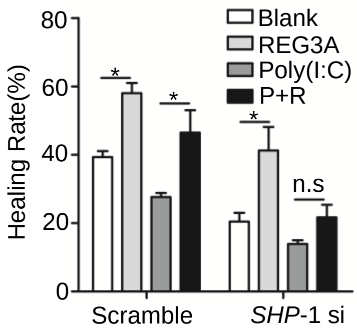

Supplementary Figure 9. SHP-1 is not involved in keratinocyte proliferation induced by REG3A. Wound re-epithelialization at 36 hours in NHEKs treated with or without 1μg ml^{-1} poly(I:C) and/or 0.03nM REG3A before or after SHP-1 was silenced. **P*<0.05. n.s. no significance. *P* values were analyzed by Two-way ANOVA. Data are the means ± s.e.m. and representative of two independent experiments.

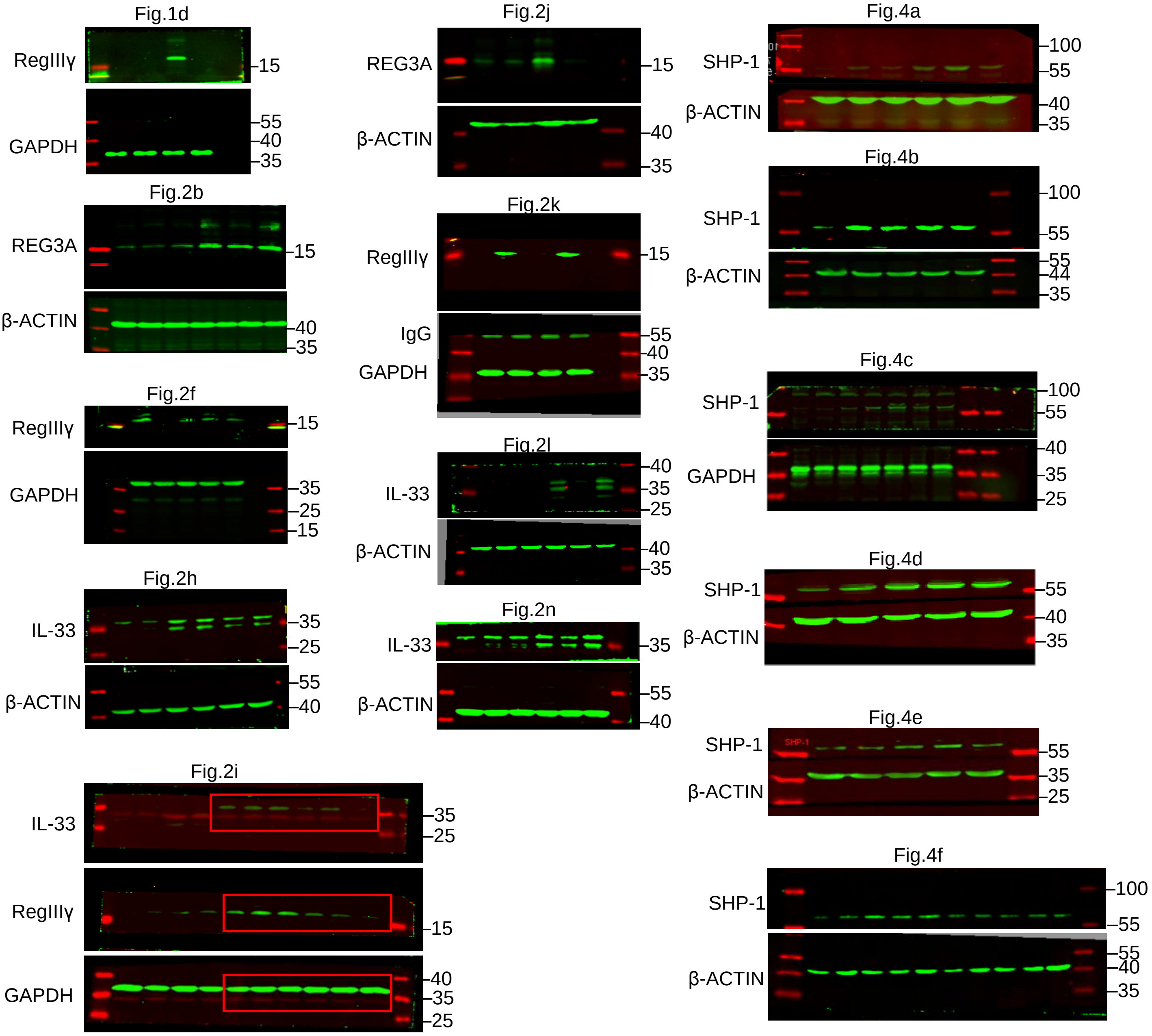

Supplementary Figure 10. Original immunoblots for indicated figures.

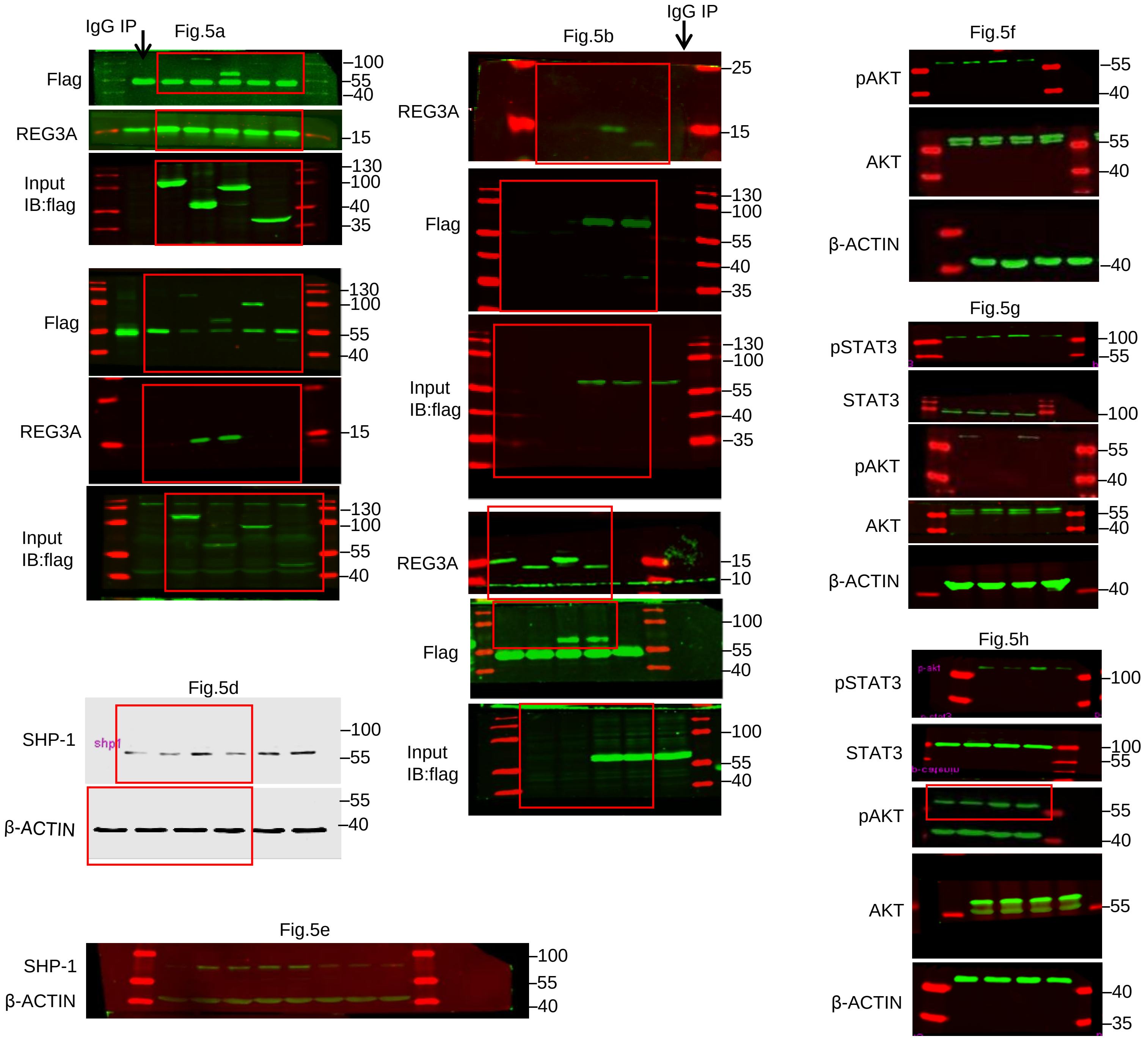

Supplementary Figure 11. Original immunoblots for indicated figures.

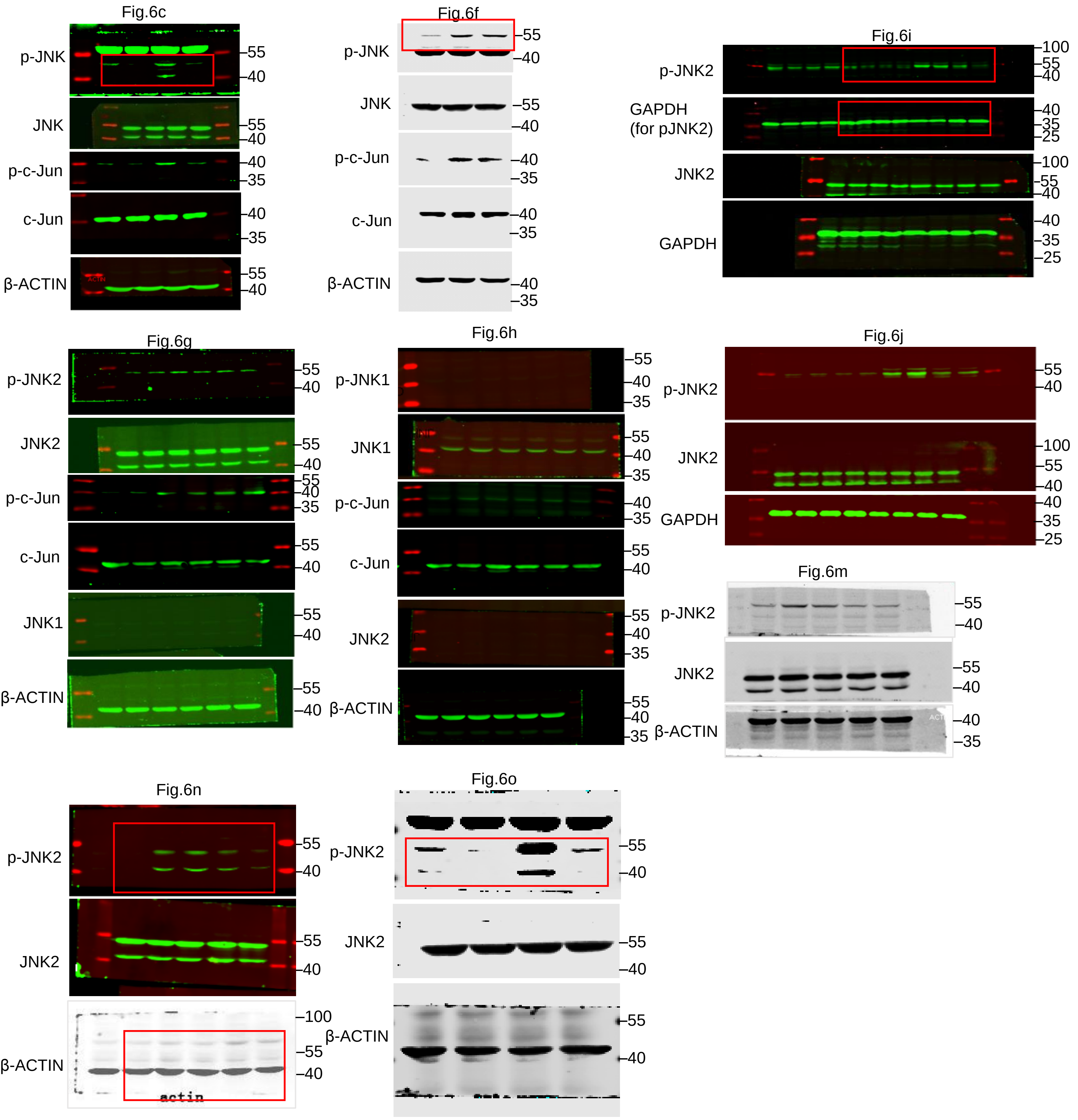

Supplementary Figure 12. Original immunoblots for indicated figures.

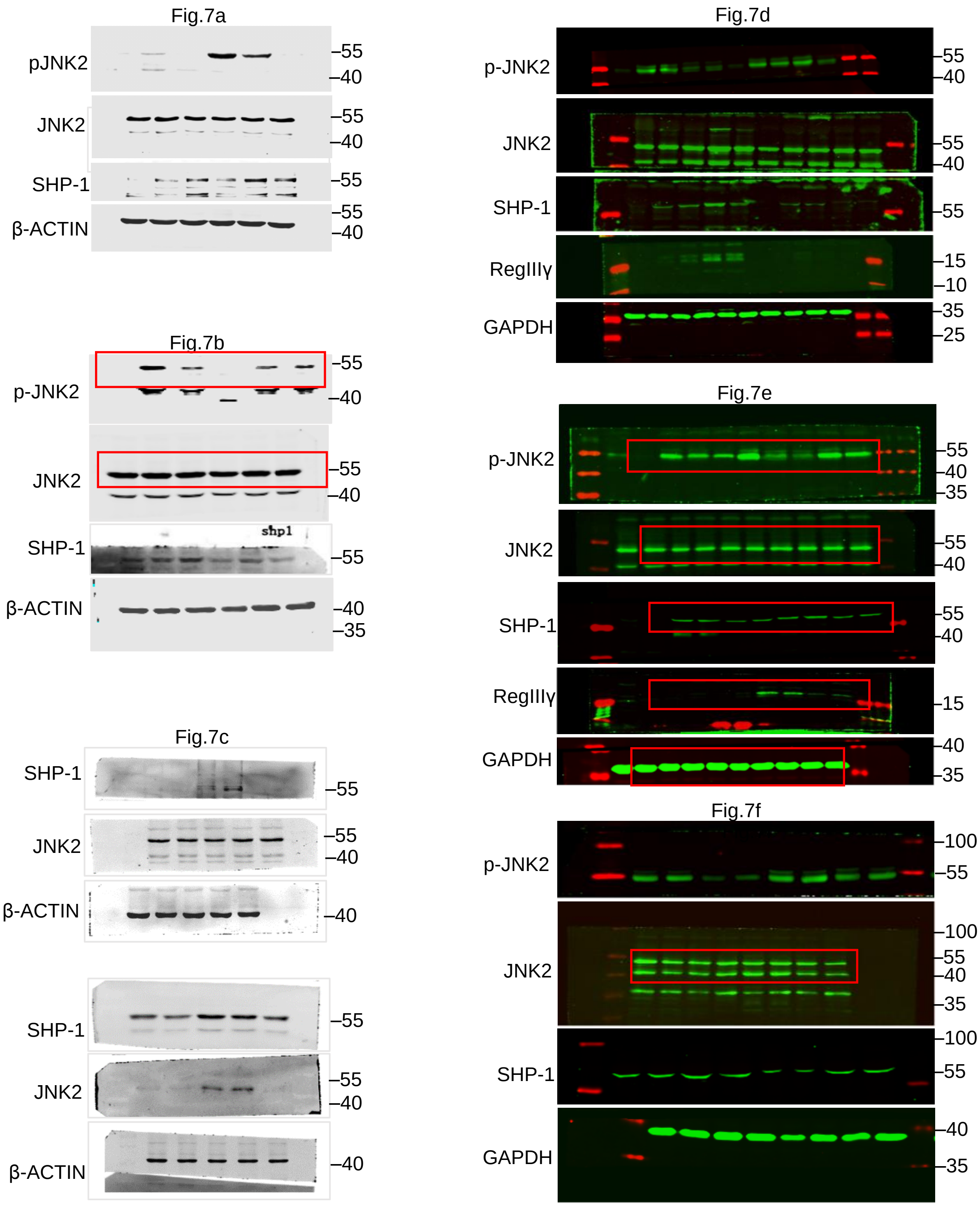

Supplementary Figure 13. Original immunoblots for indicated figures.

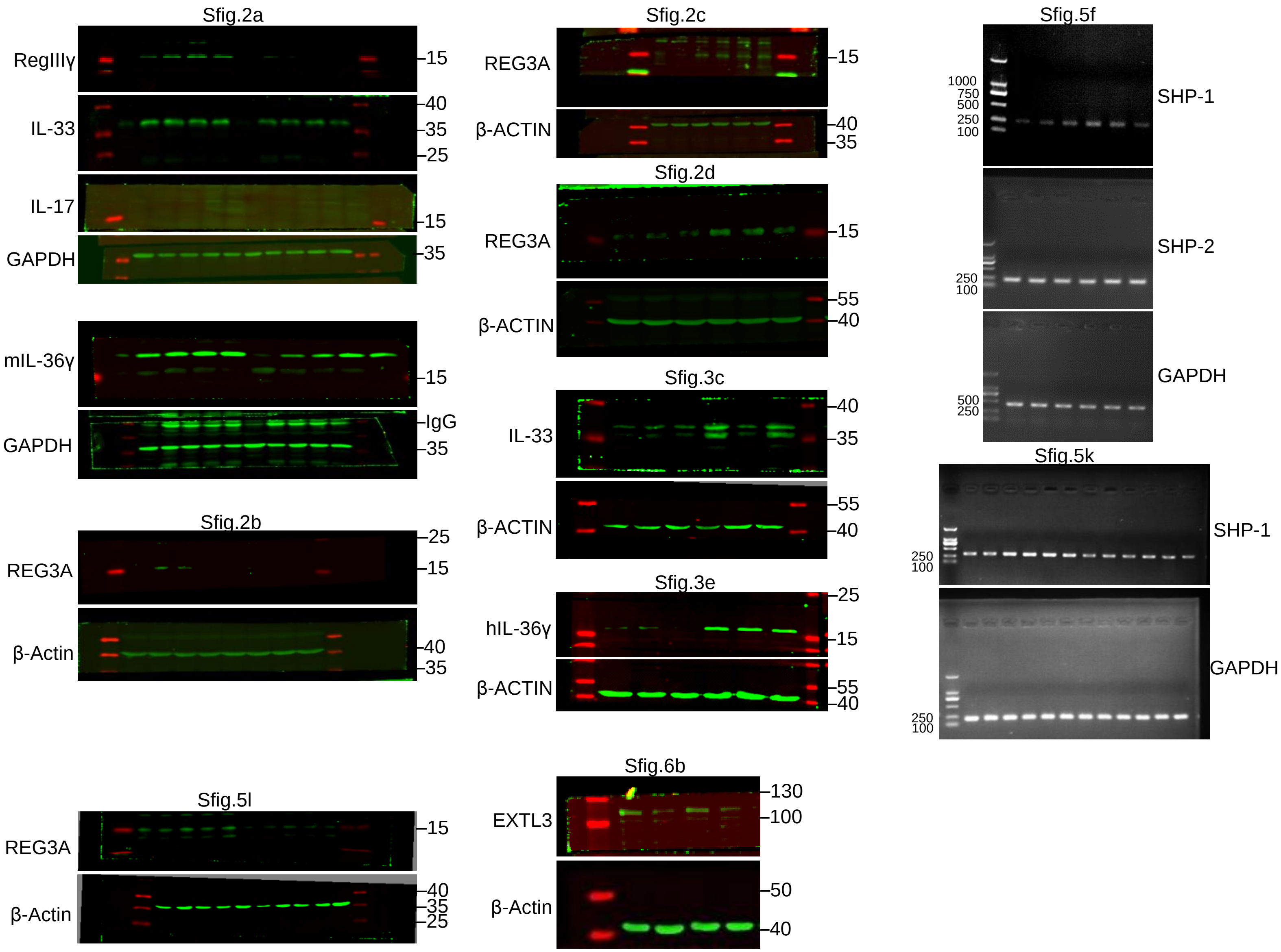

Supplementary Figure 14. Original immunoblots or agarose gels for indicated figures.

Supplementary tables

Table 1 The information of human patients

**Patients**	Normal patients with acute injury	Diabetic patients with acute injury
**Gender and age**	1. Male, 70-year-old<br>2. Female, 34-year-old<br>3. Male, 58-year-old<br>4. Female, 45-year-old<br>5. Male, 52-year-old	1. Male, 42-year-old<br>2. Male, 66-year-old<br>3. Male, 57-year-old<br>4. Female, 53-year-old<br>5. Female, 55-year-old

Table 2 Primers for constructing plasmids containing luciferase reporter of TNF-α or IL-6 promoter

	Forward	Reverse
TNF-α	GCGGTACCGCACTCGATGTACCAC	GCCTCGAGCTGGCTGGGTGTGCCAACAAC
IL-6	GCGGTACCACTTCACATCAGCTATGATGC	GCAAGCTTTCACAATCGGTTTCTTTGCTTTG
TNF-a mutant	CAGGAGACCTCTGGGGAGATTGGGTAGGAGA ATG	CATTCTCCTACCCAATCTCCCCAGAGGTCTCC
IL-6 mutant (-1739 to-1733)	GAGGCGCGTGGATGAGTTCAAGACCCGCCTG	CAGGCGGGTCTTGAACTCATCCACGCGCCTC
IL-6 mutant (-135 to-129)	GACAATCGGTGAAGAATGGCCCCAACAGC	CCTGTTGGGGCCATTCTTCACCGATTGTC
c-jun	GCGAATTCATGACTGCAAAGATGGAAAC	GCAAGCTTTCAAAATGTTTGCAACTGCTGCGT TAG

Table 3 Human REG3A, IL-33 and SHP-1 shRNA oligonucleotides

	5’	STEMP	Loop	STEMP	3’
**REG3A shRNA**					
REG3A-shRNA-1F	T	GCAGTGATGTGATGAATTA	TTCAAGAGA	TAATTCATCACATCACTGC	TTTTTTC
REG3A-shRNA-1R	TCGAGAAAAAA	GCAGTGATGTGATGAATTA	TCTCTTGAA	TAATTCATCACATCACTGC	A
REG3A-shRNA-4F	T	GTGAAGAGCATTGGTAACA	TTCAAGAGA	TGTTACCAATGCTCTTCAC	TTTTTTC
REG3A-shRNA-4R	TCGAGAAAAAA	GTGAAGAGCATTGGTAACA	TCTCTTGAA	TGTTACCAATGCTCTTCAC	A
REG3A-shRNA-5F	T	GTAACAGCTACTCATACGT	TTCAAGAGA	ACGTATGAGTAGCTGTTAC	TTTTTTC
REG3A-shRNA-5R	TCGAGAAAAAA	GTAACAGCTACTCATACGT	TCTCTTGAA	ACGTATGAGTAGCTGTTAC	A
**IL-33 shRNA**					
IL-33-shRNA-1F	CCGG	CCTTCATAATATGCACTCCAA	CTCGAG	TTGGAGTGCATATTATGAAGG	TTTTTG
IL-33-shRNA-1R	AATTCAAAAAA	CCTTCATAATATGCACTCCAA	CTCGAG	TTGGAGTGCATATTATGAAGG	A
IL-33-shRNA-2-F	CCGG	CCTGTTACTTTAGGAGAGAAA	CTCGAG	TTTCTCTCCTAAAGTAACAGG	TTTTTG
IL-33-shRNA-2-R	AATTCAAAAAA	CCTGTTACTTTAGGAGAGAAA	CTCGAG	TTTCTCTCCTAAAGTAACAGG	A
IL-33-shRNA-3-F	CCGG	GAGTGCTTTGCCTTTGGTATA	CTCGAG	TATACCAAAGGCAAAGCACTC	TTTTTG
IL-33-shRNA-3-R	AATTCAAAAAA	GAGTGCTTTGCCTTTGGTATA	CTCGAG	TATACCAAAGGCAAAGCACTC	A
**SHP-1 shRNA**					
SHP-1-shRNA-1-F	T	CTGGTGGAGCATTTCAAGATT	TTCAAGAGA	AATCTTGAAATGCTCCACCAG	TTTTTTC
SHP-1-shRNA-1-R	TCGAGAAAAAA	CTGGTGGAGCATTTCAAGATT	TCTCTTGAA	AATCTTGAAATGCTCCACCAG	A
SHP-1-shRNA-2-F	T	CGCAGTACAAGTTCATCTATT	TTCAAGAGA	AATAGATGAACTTGTACTGCG	TTTTTTC
SHP-1-shRNA-2-R	TCGAGAAAAAA	CGCAGTACAAGTTCATCTATT	TCTCTTGAA	AATCTTGAAATGCTCCACCAG	A
SHP-1-shRNA-3-F	T	CAACCCTTCTCCTCTTGTATT	TTCAAGAGA	AATACAAGAGGAGAAGGGTTG	TTTTTTC
SHP-1-shRNA-3-R	TCGAGAAAAAA	CAACCCTTCTCCTCTTGTATT	TCTCTTGAA	AATACAAGAGGAGAAGGGTTG	A

Table 4 siRNA oligonucleotides

	Forward	Reverse
EXTL3 siRNA1	CCUGCCUUUACGUGAUACUTT	AGUAUCACGUA AAGGCAGGTT
EXTL3 siRNA2	GGCUCUACAACUCCAACUAT T	UAGUUGGAGUUGUAGAGCCTT
SHP-1 siRNA1	GGUGAAUGCGGCUGACAUUTT	AAUGUCAGCCGCAUUCACCTT
SHP-1 siRNA2	CCUGGAGACUUCGUGCUUUTT	AAAGCACGAAGUCUCCAGGTT
SHP-1 siRNA3	GCAAGCACCAGGGUGACUUTT	AAGUCACCC UGGUUCUUGCTT
Negative control	UUCUCCGAACGUGUCACGUTT	ACGU GACACGUUCGGAGAATT

Table 5 PCR primers

	Forward	Reverse
REG3A	GGCACCGAGCCCAATG	GGATTTCTCTCCCATGCAAAGT
RegIIIγ	TTCCTGTCCTCCATGATCAAAA	CATCCACCTCTGTTGGGTTCA
hGAPDH	CTTAGCACCCCTGGCCAAG	TGGTCATGAGTCCTTCCACG
mGAPDH	CTTAGCCCCCCTG GCCAAG	TGGTCATGAGCCCTTCCACA
hIL-6	CAATCTGGATTCAATGAGGAGAC	TCTGGCTTGTTCCTCACTACTC
mIL-6	CTGCAAGAGACTTCCATCCAGTT	GGGAAGGCCGTGGTTGTC
hTNF-α	CCAGGCAGT CAGATCATCTTCTC	AGCTGGTTATCTCTCAGCTCCAC
mTNF-α	TCAAGGACTCAAATGGGCTTTC	TGCAGAACTCAGGAATGGACAT
hSHP-1(semi)	TGAACAAGAAGCAGGAGTC	CAGTTGGTCACAGAGTAGG
hSHP-2 (semi)	AGAGCAATGACGGCAAG	TTGGGATCACCATCGTGTAG
hTNFAIP3	TTGTGTGGCCCTCCTTCAG	CAGAACCGGCCCCTTATCA
hSARM	CCGACTCCTGGCCCTTCT	GGACATGCAGGCTGTGCTT
hTRAF1	AGCCTCTGGATGGTGCAATAAT	TTCCGGCCTGACCTAAGCT
		Fluorogenic probe and primers:
RegIIIγ	in *Lepr*$^{db/db}$ mice	Mm00441127_m1

Supplementary Methods

Animals

Control (*Lepr*$^{db/+}$) mice and mice homozygous for the diabetes spontaneous mutation (*Lepr*$^{db/db}$) (Stock No: 000642) were purchased from Jackson Laboratory. NOD mice (30 weeks, female) were purchased from SLAC Laboratory Animal Center. All mice were housed and bred in specific pathogen-free conditions in the animal facilities in East China Normal University and Loyola University Chicago. All mouse experiments were approved by East China Normal University Animal Care and Use Committee and the Loyola University Chicago IACUC committee. All surgeries were performed under anesthesia and all efforts were made to minimize suffering. For all mouse studies we performed preliminary experiments to determine requirements for sample size. Mice were assigned randomly to experimental groups but not performed in a blinded fashion.

Reagents

Poly(I:C) was purchased from InvivoGen. Streptozotocin (STZ) and inhibitors including bay11, PD98059, SB202190, LY294002, SP600125, AKT1/2 inhibitor, and SSG were purchased from Sigma; Wortmanin was from Calbiochem; Stat3 inhibitor VI (S3I-201) and AGE Inhibitor (Aminoguanidine, AG) were from Santa Cruz Biotechnology.

Antibodies used for Immunoblot were listed as the following: anti-β-actin (Sigma, A5441), anti-GAPDH (CST, 5174S), anti-mouse RegIIIγ (ABclonal Biotechnology, WG-00077D-K39), anti-human REG3A (SHANGHAI IMMUNOGEN BIOLOGICAL TECHNLOGY&CO., LTD, A101122-LZ0098), anti-human IL-33 (R&D, AF4810), anti-mouse IL-33 (R&D, AF3626), anti-human SHP-1 (R&D, MAB18781-427043),

anti-mouse SHP-1 (CST, 3759S), anti-human EXTL3 (R&D, MAB2635), anti-p-JNK (CST, 9251S), anti-p-AKT (CST, 4060S), anti-p-STAT3 (CST, 9131L), anti-p-p38 MAPK (CST, 4511S), anti-p-β-catenin(CST, 9566), anti-p-c-Jun (CST, 3270S), anti-AKT (CST, 4691S), anti-STAT3 (CST, 9132), anti-β-catenin (CST, 9562S); anti-JNK2 (Sino Biological, 10745-R004), anti-JNK1 (Abcam, ab199380), anti-c-Jun (CST, 9165S) and anti-IL-17 (R&D, MAB421).

Plasmids

Human *SHP-1*, S*HP-1C453S, c-Jun, EXTL3,* and its variants were cloned into mammalian expression vector named pcmv-tag2B. All lentiviral plasmids were constructed in a modified **pll3.7** vector. cDNAs including human *IL-17*, human *IL-36γ*, mouse *IL-33*, human *IL-33*, mouse *RegIIIγ*, human *REG3A* and its variants were amplified by PCR and then subcloned into **pET-32a** for expression in *E.coli*. All the information of these DNA sequences was from NCBI database.

Expression and purification of recombinant proteins

Plasmids expressing human IL-17, human IL-33, mouse IL-33, human IL-36γ, REG3A, RegIIIγ or REG3A variants were transformed into *Escherichia coli* BL21(DE3), respectively. After 1mM isopropyl β-D-1-thiogalactopyranoside (IPTG) induction for 4h, bacteria were collected and sonicated. REG3A, RegIIIγ or REG3A variants was expressed in the form of inclusion bodies and were solubilized by denaturing buffer and refolded by refolding buffer. After dialysis for 24h, proteins were purified by HiTrp ion exchange columns according to the manufacturer’s instructions (GE Healthcare). For IL-17, IL-36γ and IL-33 expression and purification, IL-17, IL-36γ and IL-33 containing hexahistidine at N-terminus were purified

by a Ni^{2+}-nitrilotriacetic acid resin (NTA) column after 1mM IPTG induction for 4h, and went through the Hitrap Q FF column to remove the endotoxin according to the manufacturer's instructions (GE Healthcare).

Excisional wound model

Dorsal mouse fur from age-matched control and NOD mice (30 weeks, female), or ($Lepr^{db/+}$) mice and mice homozygous for the diabetes spontaneous mutation ($Lepr^{db/db}$) (8–10 weeks, male) was removed by plucking or using chemical depilation (Veet) under isoflurane anesthesia. Excisional dorsal skin wounds were made with a 5 mm or an 8mm sterile biopsy punch. Mice were euthanized on day 3, 5, 7 post-wounding, and 5mm skin surrounding wound edges was used for immunohistochemical analysis, 2mm skin surrounding wound edges was collected for qPCR.

Quantitative RT-PCR

Total RNA was extracted from mouse wound homogenates using TRIzol® reagent (Life Technologies) following the manufacturer's introduction. Complementary DNA was synthesized using iScript™ cDNA Synthesis Kit (Bio Rad) or cDNA Reverse Transcriptase kit (Roche). Quantitative RT-PCR-specific primers of RegIIIγ are shown in Supplementary Table 5. Quantitative RT-PCR was performed in triplicate using TaqMan® Universal PCR Master Mix (Life Technologies) or SYBR green master mix (Roche) on a StepOnePlus™ Real-Time PCR System (Applied Biosystem). Samples with low yield of RNA were pre-determined and excluded.

Semi-quantitative RT-PCR

Total RNA was extracted from cells using TRIzol® reagent (Life Technologies) following

the manufacturer’s introduction. Equal amounts of total RNA from each sample were reversed using cDNA Reverse Transcriptase kit (Roche). The RT reaction was then amplified for house-keeping gene cDNA (GADPH) and specific cDNA of interest using gene-specific primers (Table 5). After PCR, same volumes of reaction products were electrophoresed on a 1% agarose gel. Uncropped images of the agarose gels for Supplementary Fig.5 are shown in Supplementary Fig.14.
